# Heparin interferes with the uptake of liposomes in glioma

**DOI:** 10.1016/j.ijpx.2023.100191

**Published:** 2023-06-21

**Authors:** Thomas S. van Solinge, Kristina Pagh Friis, Killian O'Brien, Romy L. Verschoor, Jeroen van Aarle, Arnold Koekman, Xandra O. Breakefield, Pieter Vader, Raymond Schiffelers, Marike Broekman

**Affiliations:** a Department of Neurology and Center for Molecular Imaging Research, Department of Radiology, Massachusetts General Hospital and NeuroDiscovery Center, Harvard Medical School, Boston, MA 02114, USA; b Department of Neurosurgery, Leiden University Medical Center, Leiden, the Netherlands; c Department of Neurosurgery, University Medical Center, Utrecht, the Netherlands; d Department of Clinical Chemistry and Haematology, University Medical Center Utrecht, Utrecht, the Netherlands; e CDL Research, University Medical Center Utrecht, Utrecht, the Netherlands; f Department of Neurosurgery, Haaglanden Medical Center, The Hague, the Netherlands

**Keywords:** Liposomes, Heparin, Glioma, Glioblastoma, Uptake, Delivery

## Abstract

In glioblastoma, a malignant primary brain tumor, liposomes have shown promise in pre-clinical and early phase clinical trials as delivery vehicles for therapeutics. However, external factors influencing cellular uptake of liposomes in glioma cells are poorly understood. Heparin and heparin analogues are commonly used in glioma patients to decrease the risk of thrombo-embolic events. Our results show that heparin inhibits pegylated liposome uptake by U87 glioma and GL261 cells in a dose dependent manner *in vitro,* and that heparin-mediated inhibition of uptake required presence of fetal bovine serum in the media. In a subcutaneous model of glioma*,* Cy5.5 labeled liposomes could be detected with *in vivo* imaging after direct intra-tumoral injection. *Ex-vivo* analysis with flow cytometry showed a decreased uptake of liposomes into tumor cells in mice treated systemically with heparin compared to those treated with vehicle only.

## Graphical abstract

**Figure:**
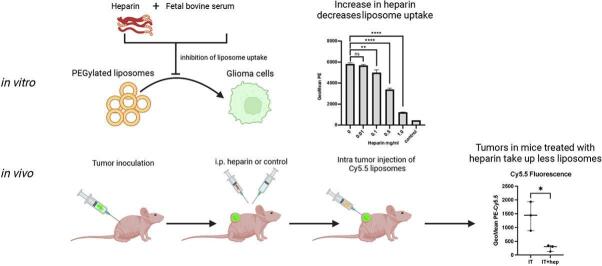

## 1 Introduction

Liposomes are small, nanosized, spherical lipid based particles, which consist of one or multiple lipid bilayers formed by self-assembly from a mixture of phospholipid(s) and cholesterol. Their size can range from 30 nm (nm) to several micrometer (μm), and they are highly suitable to transport aqueous or lipid drugs.(Akbarzadeh et al., 2013) Furthermore, liposomes are highly customizable, allowing for a targeted approach to therapy, such as the addition of lipid-anchored polyethylene glycol (PEG).(La-Beck et al., 2012; Gabizon et al., 2016) Initial excitement for the use of liposomes in oncology has waned in recent years, as promising pre-clinical studies have struggled to translate these results to a clinical setting.(Crommelin et al., 2020)

Liposomal delivery of therapeutics has shown major promise in pre-clinical studies regarding malignant glial tumors.(Li et al., 2020; Amarandi et al., 2022) Glioblastoma and other malignant gliomas are derived from glial cells and affect around 19.800 people each year in the US alone.(Ostrom et al., 2019) Survival remains abysmal, with few improvements in patient outcome since the introduction of the Stupp protocol in 2005.(Stupp et al., 2005) One of the challenges for systemic therapy for glioblastoma is that the interior milieu of the brain is tightly regulated by the blood-brain barrier (BBB), requiring high systemic dosing to reach therapeutic concentrations in the CNS(Abbott et al., 2010). Pre-clinical studies have shown liposomes to be able to cross the BBB, and improve delivery and efficacy of various compounds in brain tumors.(Li et al., 2020; Siegal et al., 1995; Li et al., 2021) The field is in continuous development, with different formulations of liposomes (including of size, surface charge, surface pKa, and PEG content) being tested to improve crossing the BBB and uptake by glioma cells.(Li et al., 2020; Joshi et al., 2016) Generally, two major categories of liposomes are being tested: PEGylated, untargeted liposomes and more recently liposomes specifically designed to target glioma cells. While untargeted doxorubicin-loaded liposomes showed great efficacy in mouse models,(Li et al., 2021; Lakkadwala et al., 2019; Grafals-Ruiz et al., 2020) Phase I and II trials with untargeted liposomes have been disappointing.(Ananda et al., 2011; Beier et al., 2009) To improve efficacy, a wide range of targeted liposomes are being developed. Recently, EGFR targeting PEGylated liposomes containing doxorubicin were shown to be able to hone to tumor tissue in nine glioblastoma patients, but only in areas where the BBB was disrupted in a Phase I trial.(Kasenda et al., 2022) Adding glutathione (GSH) to PEGylated liposomes utilizes naturally present GSH-transporters to cross the BBB and showed excellent tumor response in mice.(Gaillard et al., 2014) A Phase I/IIA trial showed good safety(Brandsma et al., 2014) and further studies are currently being done in patients with breast cancer brain metastases (NCT01818713). Improving liposome delivery *via* ultrasound-mediated BBB disruption is being investigated as well.(Song et al., 2021) One of the caveats in our knowledge of liposomes and glioblastoma is that while the pharmacokinetics and BBB-crossing abilities of PEGylated liposomes are relatively well understood,(Wang et al., 2020; Gabizon et al., 2003) the factors influencing cellular uptake of these liposomes are not.

Patients suffering from glioblastoma are at increased risk of thrombo-embolic events and are often treated with heparin or heparin analogues.(Perry, 2012) Usually, a prophylactic dose of low-molecular-weight heparin is administered around neurosurgical procedures, and unfractionated heparin is used when thrombo-embolic complications occur. Heparin has been shown to interfere with uptake of extracellular vesicles (EVs) in glioma cell lines,(Atai et al., 2013) and cationic liposomes in HeLa cells,(Champanhac et al., 2021) but its effects on PEGylated liposomes as used in clinical glioblastoma trials are unknow.

In this study, we aimed to evaluate various factors influencing cellular uptake of PEGylated liposomes in glioma. We found that heparin has an inhibitory effect on cellular uptake of PEGylated liposomes *in vitro*, dependent on presence of fetal bovine serum (FBS)*.* We further studied this effect *in vivo* and found heparin to decrease uptake after direct tumor injection.

## 2 Materials and methods

### 2.1 Cell culture

Human primary glioblastoma cell line U87 was acquired from ATCC (ATCC HTB-14, Manassas, VA, USA). Cells were cultured in Dulbecco's Modified Eagle's Medium (DMEM) (Mediatech, Manassas, USA) supplemented with 10% fetal bovine serum (FBS) (Atlanta Biologicals, R&D systems, Minneapolis, MN, USA) and 1% penicillin/streptomycin (P/S) (Mediatech, Manassas, USA). Cells were grown at 37 °C in a 5% CO_2_ humidified atmosphere. For imaging and mice studies, cells were transduced with a lentiviral vector expressing Fluc-IRES-eGFP under a CMV promotor.(Tannous et al., 2005)

### 2.2 Liposomes

To prepare liposomes, appropriate amounts of dipalmitoylphosphatidylcholine (Lipoid GmbH, Ludwigshafen am Rhein, Germany), cholesterol (Sigma-Aldrich, Germany), and poly(ethylene glycol) 2000-distearoylphosphatidylethanolamine (Lipoid GmbH), in a molar ratio of 65:30:5, were dissolved in chloroform in a round-bottom flask. L-α-phosphatidylethanolamine-N-lissamine rhodamine B sulfonyl (Rho-PE) (Avanti Polar Lipids), was added at 0.1 mol% for fluorescent labeling. A lipid film was prepared under reduced pressure on a rotary evaporator and dried under a stream of nitrogen. Liposomes were formed by rehydration of the lipid film with phosphate-buffered saline (PBS, pH 7.4), to a final concentration of 10 mM total lipid (TL). Liposome size was reduced by multiple extrusion steps (Lipex high pressure extruder, Northern Lipids) using Whatman Anodisc inorganic membranes (Sigma-Aldrich) with a pore size of 100 nm. Total lipid content of the liposomal dispersion was determined with a phosphate assay of the organic phase after extraction of liposomal preparations with chloroform, according to Rouser et al.(Rouser et al., 1969)

Nanosight Tracking Analysis (NTA) (Malvern Instruments, Malvern, UK) revealed a liposome concentration of 3.0 × 10^8^ per μL and an average liposome size of 117 nm. The Cy5.5 liposomes used in the animal experiments were created *via* the same procedure, with 0.2 mol% Cy5.5-DSPE instead of Rho-PE being added during preparation. NTA showed a concentration of 5.0 × 10^8^ liposomes per μL with an average size of 160 nm.

### 2.3 *In vitro* evaluation of liposomal uptake

For evaluation by flow cytometry analysis, U87 cells were plated in 24-well plates (10^5^ cells/well) and cultured for 24 h. Culture medium was removed, and cells were incubated with liposomes diluted in conditioned medium (with or without 10%FBS) and increasing concentrations of heparin (heparin sodium 1000 USP Units/mL, Fresenius Kabi USA, Lake Zurich, IL).

A total of 3.0 × 10^9^ liposomes was used per well. After four hours of incubation, cells were collected by centrifugation (300 ×*g*/5 min). The supernatant was removed, and cells were resuspended in PBS for flow cytometry analysis using the Fortessa-X20 flow cytometer (BD Biosciences). Further analysis was performed using FlowJo software (version 10.7.1, Becton & Dickinson company, Franklin Lakes, NJ, USA). Statistical analysis was performed using GraphPad Prism software (version 8.4.3, San Diego, USA). Statistical significance between samples was determined using an unpaired *t-*test. Significance was set to a value of 0.05.

### 2.4 Cell viability assay

50.000 U87 cells were plated in a 24 well plate and incubated under standard conditions for 24 h. Media was removed and cells were incubated in DMEM with or without FBS, with increasing concentrations of heparin for four hours. For cell viability, Cell Counting Kit-8 (CCK-8) (Dojindo, Rockville, MD, USA) was used. The CCK-8 reagent was added to the wells, incubated for 2 h and absorbance was measured on a plate reader: Synergy H1 (Biotek Instruments Inc., Winooski, VT, USA). To measure cell death, the LDH Assay Kit (Dojindo) was used according to the manufacturer's protocol, with absorbance measured on the plate reader.

### 2.5 Imaging

U87 cells were stably transduced with a lentivirus vector encoding an expression cassette for palmitoylated GFP to visualize the plasma membrane.(Zacharias et al., 2002) Cells were plated on coverslips coated with 0.01% poly-l-lysine and cultured for 24 h. Cells were then incubated with rhodamine-liposomes with or without heparin (0.5 mg/mL). As a control, cells were incubated in media only. After four hours of incubation, cells were washed three times with double filtered PBS and fixed with 4% formaldehyde. Coverslips were transferred to microscope glasses with the use of gold antifade reagent with DAPI (Thermofisher Scientific). Slides were analysed with fluorescent imaging using the B*Z*-X microscope (Keyence, Itasca, IL, USA). ImageJ 2.0.0v software was used to process the images and create Z-stacks.

### 2.6 Mice studies

Male and female athymic nude mice (Charles River Laboratories Wilmington, MA USA) aged between 8 and 12 weeks, were used for all experiments. Animals were housed at the animal facility of the Massachusetts General Hospital under standard laboratory conditions, with free access to water and food. For tumor inoculation, 1.0 × 10^6^ U87-Fluc-GFP cells were suspended in 50μLSodium-Chloride 0.9% and mixed with 50μLMatrigel (Corning, Corning NY, USA). Mice were anesthetized with isoflurane, and the cell were injected on the right flank. After injection of tumor cells, mice were monitored daily to assess health, appearance, and behavior. All experiments were approved under IACUC protocol 2009 N000054.

### 2.7 Bioluminescence imaging

100 μL D-Luciferin (Thermo-Fischer) (25 mg/ml in saline) was injected intra-peritoneally in isoflurane-anesthetized mice. After five minutes, mice were imaged using the IVIS (*In Vivo* Imaging System) Spectrum connected to an X GI-8 Anesthesia System (PerkinElmer, Waltham, MA USA). Bioluminescence was expressed as Total Flux per second.

### 2.8 Heparin administration and liposome injection

Mice were divided into two groups with equal tumor size based on bioluminescence. One group was injected intraperitoneally with 10 IU of heparin‑sodium in 100 μL0.9% Sodium-Chloride every 12 h for 3 days. The control group was injected with 100 μL0.9% Sodium-Chloride at similar intervals. On day 3, all mice were injected intra-tumorally with 5 × 10^9^ liposomes.

Liposomes were diluted in 50 μL0.9% Sodium-Chloride and injected in five different areas of the tumor. After four hours, mice were euthanized, and the tumors removed for flow cytometric analysis.

### 2.9 Sample processing and flow cytometry

Tumors were removed from deeply anesthetized animals, digested and prepared for flow using the Tumor Dissociation Kit, mouse (Miltenyi Biotec, Bergisch Gladbach, Germany) per manufacturers protocol. In brief, tumors are cut into small pieces with scissors and placed into C-tubes (Miltenyi Biotec). Enzymes are added and the tissue further minced with the gentleMACS dissociator (Miltenyi Biotec). The samples were then incubated at 37 °C for 40 min on a rotator, and afterwards minced in the dissociator. The samples were run through a 70 μm filter and washed with Dulbecco's PBS (DPBS) without magnesium or calcium +0.5% bovine serum albumin (BSA) and 2 mM ethylenediaminetetraacetic acid (EDTA). 4′,6-diamidino-2-phenylindole (Dapi) was added as a live/dead cell marker. Flow cytometry was done on a BD LSRFortessa cell analyser (Becton Dickinson (BD), Franklin Lakes, NJ), and analysis was performed using FlowJo version 10 (FlowJo, Ashland, OR).

## 3 Results

### 3.1 Uptake of liposomes is inhibited by heparin

**Fig. 1:**
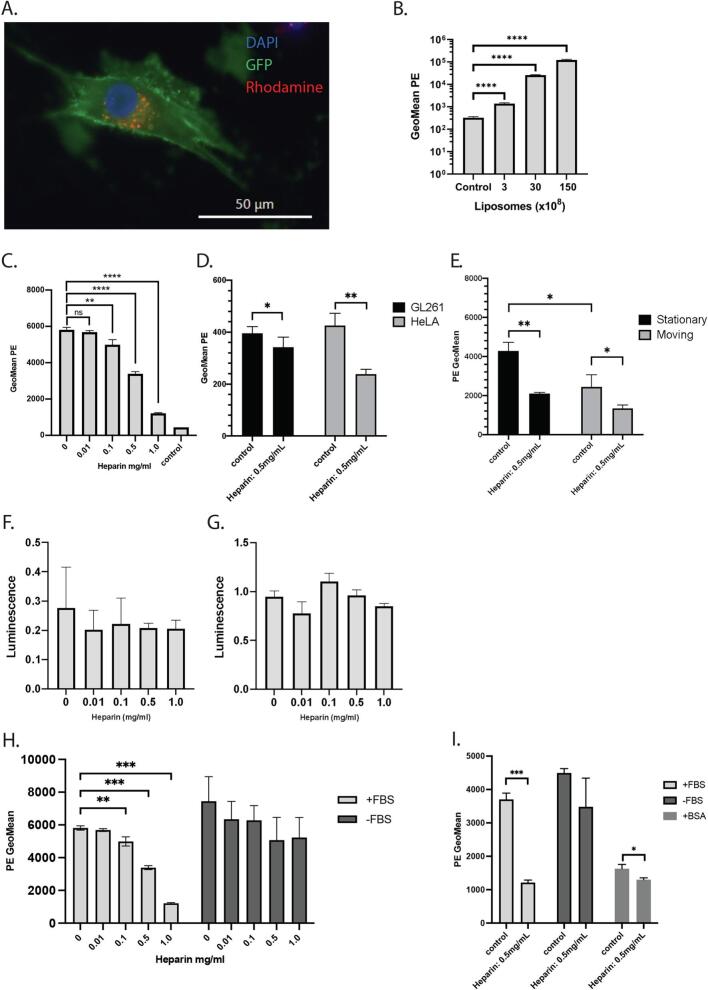
Uptake of liposomes and effects of heparin in glioma. A) U87-palmGFP cell after 4 h incubation with rhodamine labeled liposomes. B) Dose-dependent uptake of liposomes in U87 cells. Cells were incubated with liposomes for 4 h. Three replicates each, measured by flow cytometry. C) Heparin decreases liposome uptake in U87 cells in a dose dependent manner in medium with serum. Three replicates, measured by flow cytometry. ns: not significant, **: *p* < 0.01, ****:*p* < 0.0001. D) Decrease in liposome uptake by HeLa cells and GL261 cells upon heparin incubation. *: *p* < 0.05, **: *p* < 0.01. E) Unattached cells show less uptake of liposomes *in vitro*. Heparin decreases uptake in similar fashion whether cells are allowed to attach (stationary) or not (floating). *: p < 0.05, **: p < 0.01. F) Increased doses of heparin do not affect cell viability as measured by Cell Counting Kit-8. G) Increased doses of heparin do not affect cell death as measured by LDH Assay Kit. H) Absence of Fetal Bovine Serum (FBS) diminishes the inhibitory effect of heparin on liposomal uptake. **: p < 0.01, ***: *p* < 0.001. I) Adding 45 mg/ml Bovine Serum Albumin restores the inhibitory effect of heparin, although uptake overall is diminished as compared to FBS and non-FBS incubated U87 cells. *: p < 0.05, ***: p < 0.001.

To demonstrate that GB cells take up liposomes, we incubated U87 cells with liposomes loaded with a fluorescent dye, Rhodamine. U87 cells showed strong uptake of liposomes (Fig. 1A) in a dose-dependent manner (Fig. 1B). *Z*-stack imaging showed internalisation of the liposomes in the cell (*Supplementary movie A).*

Next, we incubated U87 cells with liposomes and increasing dosages of heparin for four hours. With flow cytometry we were able to show significant uptake inhibition by heparin from 0.1 mg/ml (independent student-*t-*test, *p* < 0.001) (Fig. 1C *and Supp.* Fig. 1A). Increases in heparin were significantly correlated with inhibition of uptake: R: 0.9895, *p* < 0.01 (*Supp.* Fig. 1B). The effect of heparin could be observed in liposomes dosages as low as 3.0 × 10^8^ and as high as 3.0 × 10^10^ (*Supp.* Fig. 1C*)*. In GL261 cells and HeLa cells, we observed a similar decrease in uptake of liposomes upon incubation with 0.5 mg/ml heparin, although the effect was less pronounced (independent t-test: GL261, *p* = 0.02, HeLa, *p* < 0.01. Fig. 1D*).*

### 3.2 Heparin inhibition is diminished in absence of fetal bovine serum (FBS)

To elucidate the mechanism of uptake inhibition by heparin, we explored various pathways in which heparin could interfere with uptake. Heparin is known to have anti-adhesive effects in cells.(Xu and Dai, 2010) Upon incubation with heparin, we saw rapid detachment of U87 cells from the wells. This effect was observed at dosages of 0.1 mg/ml and higher (*Supp.* Fig. 1D). To evaluate whether detachment of the cells would interfere with the uptake of liposomes, we incubated 50.000 U87 cells with heparin and liposomes in non-coated plastics on a gentle shaker to prevent adhesion. Media was removed after 4 h and no trypsinization was performed to exclude cells that might have adhered to the wells. Overall, uptake of liposomes was significantly reduced compared to the cells that were allowed to adhere overnight (student *t*-test, *p* = 0.04), but heparin still showed a significant reduction in uptake, student *t*-test, p = 0.04 (Fig. 1E). To assess whether heparin affected the viability of the cells, we performed a cell viability assay and a cell death assay after four hours of incubation with heparin (Fig. 1F,G). Heparin showed no effect on viability and cell death with increasing doses of heparin under both conditions. Previously, Atai et al(Atai et al., 2013) observed clustering of EVs upon increasing doses of heparin with transmission electron microscopy. We incubated liposomes with and without heparin for four hours and analysed liposome size *via* NTA (*Supp.* Fig. 1E,F*)*. Mean liposome size did not vary significantly with liposome size measuring 104 nm with a standard deviation (SD) of 34.4 nm, while heparin incubated liposomes had a mean size of 92.2 nm, SD 35.6 nm (student's t-test, *p* = 0.6) . This suggest that the liposomes did not cluster as a result of heparin exposure under the conditions used in these experiments.

We then explored whether the inhibitory effect of heparin depended on additional components in the media. To this aim, we incubated U87 cells with 3 × 10^9^ rhodamine-labeled liposomes and increasing concentrations of heparin with or without the presence of 10% FBS. In cells incubated without FBS, the inhibitory effect of heparin on liposome uptake was diminished (Fig. 1H*)*. There was a slight, but not significant, increase in uptake of liposomes after incubation without FBS. Cell viability and cell death did not differ between U87 cells incubated with or without FBS under these conditions (*Supp*. Fig. 1G,H*).* PEGylated liposomes are known to react with serum proteins.(Palchetti et al., 2016) To evaluate whether the presence of bovine proteins in the FBS played a role in the heparin-liposome interaction, we incubated U87 cells with 45 mg/ml BSA, comparable to the protein concentration in FBS, and compared uptake of liposomes in cells incubated with or without FBS (Fig. 1I*)*. Although overall uptake was diminished in cells incubated with BSA, a small but significant inhibitory effect of heparin on liposome uptake could be observed.

Overall, heparin seems to rely on multiple mechanisms to inhibit uptake. Detachment of cells plays a role, as does the interaction between heparin and serum proteins present in the media and on the cells. Different than EVs, heparin does not seem to induce aggregation of liposomes in our set-up.

### 3.3 Heparin decreases uptake of liposomes *in vivo*

**Fig. 2:**
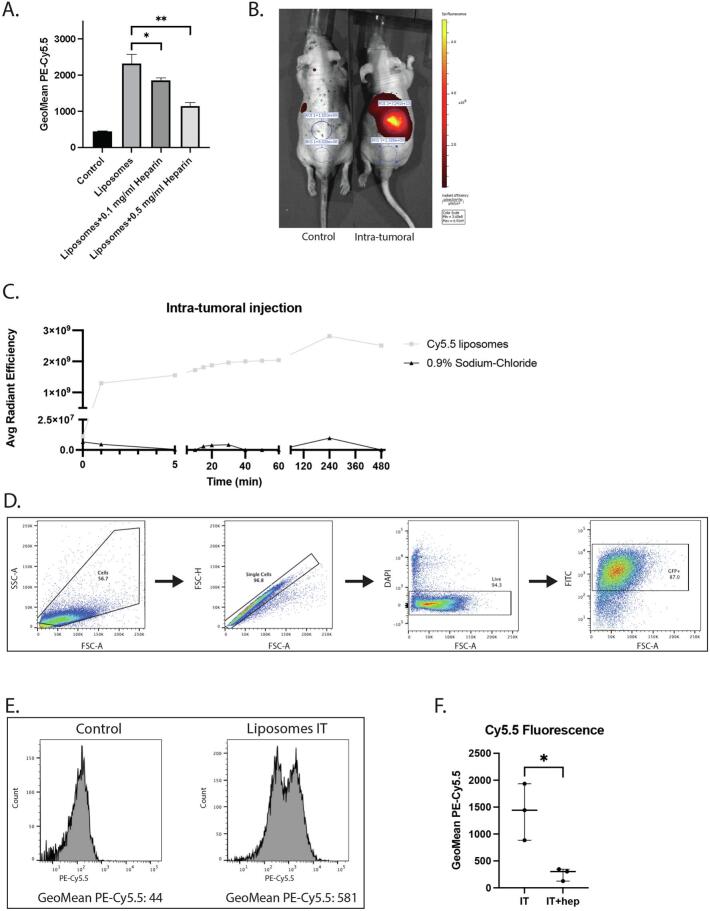
Heparin inhibits uptake of liposomes *in vivo*. A) Heparin decreases Cy5.5 liposome uptake in U87 cells in a dose dependent manner in medium with serum. Three replicates, measured by flow cytometry. *: *p* < 0.05, **: *p* < 0.01. B) IVIS image of fluorescent signal 4 h after injection of control or liposomes intra-tumorally. C) Uptake of liposomes into tumors over time *in vivo* as measured by IVIS imaging. Cy5.5 labeled liposomes were intra-tumorally (IT). PBS was injected as control. D) Flow gating for detection of liposomal uptake *in vivo*. Cells were selected based on forward- and side-scatter. DAPI was used as live-dead marker. GFP positive tumor cells were selected. E) Histograms comparing signal between GFP positive cells in control and intra-tumoral (IT) injection. F) Geometric Mean of Cy5.5 signal in GFP positive cells in intra-tumorally (IT) injected mice, who received either heparin or control. *: *p* < 0.05.

To assess the inhibitory effect of heparin *in vivo*, we loaded liposomes with Cy5.5, a near-infrared dye, to facilitate tracking *in vivo* with IVIS(Allijn et al., 2017). We confirmed that heparin inhibited uptake of these liposomes in similar fashion as the Rhodamine labeled liposomes (Fig. 2A). To evaluate the optimal time and method for evaluation of uptake, we implanted U87-Fluc-GFP tumor cells subcutaneously in three athymic nude mice, and after three weeks injected either saline or 5.0 × 10^9^ liposomes intra-tumorally, tracking the signal over 8 h with IVIS (Fig. 2B). Cy5.5 fluorescent signal could be detected in the tumor, plateauing 4 h after injection (Fig. 2C). To evaluate if the liposomes were taken up by the tumor cells, or remained extracellular after injection, we performed flow cytometry on dissociated cells from subcutaneous injected tumors in mice which had received liposomes intra-tumorally, or saline as a control. We selected tumor cells by GFP signal and measured the geometric mean of Cy5.5 fluorescence in these cells as a measure of liposomal uptake (Fig. 2D,E). As we were primarily interested in the effect of heparin on the uptake of liposomes by tumor cells, and less on the effects on biodistribution in general, we opted to use this intra-tumoral approach as compared to an intravenous approach.

Six athymic nude mice were subcutaneously injected with U87-Fluc-GFP tumor cells on the right flank. After two weeks, tumor size was estimated with IVIS imaging, and the mice stratified into two groups of similar average tumor size (*Supp.* Fig. 2A*)*. One group received 10 IU of heparin in 50 μL0.9% Sodium-Chloride twice daily intra-peritoneally, which mimics therapeutic heparin doses in patients,(Li et al., 2011) while controls received 50μLSodium-Chloride 0.9% twice per day. Heparin was given for two days prior to liposome injection to ensure adequate levels in the mice. On day 3, 5.0 × 10^9^ liposomes were injected intra-tumorally in 50 μL0.9% Sodium-Chloride one hour after heparin injection. After four hours, mice were euthanized and the tumors removed and processed for flow cytometry. There was no relation between tumor size, as measured by IVIS, and liposomal uptake, as measured by Cy5.5 signal in flow (*Supp.* Fig. 2B*).* Mice that had received heparin showed significantly less uptake of Cy5.5 liposomes compared than those who had not (Fig. 2F), indicating an inhibitory effect on liposome uptake in U87 cells in this *in vivo* model.

## 4 Discussion

Our findings show that liposomes are rapidly taken up by glioma cells in a dose-dependent manner. However, heparin inhibits this uptake from concentrations of 0.1 mg/ml onwards but only in the presence of serum. Absence of serum increased uptake of liposomes in general, and limited the inhibitory effects of heparin. *In vivo*, intra-peritoneal injections of therapeutic heparin doses decreased the uptake of intra-tumoral delivered liposomes in orthotopic human U87 gliomas *in vivo*.

The effects of heparin on uptake of nanocarriers have come under scrutiny, as glioblastoma patients, and cancer patients in general, are at high risk for thrombo-embolic events and are often treated with heparin and heparin-analogues.(Perry, 2012) *In vitro,* uptake of positively charged liposomes has been found to be influenced by presence of heparin in HeLa cells, monocytes, and macrophages, while uptake of negatively charged particles was not influenced by heparin.(Champanhac et al., 2021) However, most liposome formulations used in glioblastoma are neutral or negatively charged(Amarandi et al., 2022) and are often PEGylated to prevent opsonization and increase circulation time in the bloodstream.(Kolate et al., 2014) In this paper, we first describe the effects of heparin in glioma cell lines with PEGylated liposomes, which have been used in various clinical glioblastoma trials.(Ananda et al., 2011; Beier et al., 2009)

Previously, Atai et al. showed that heparin inhibits uptake of EVs in glioma cells *in vitro.(*Atai et al., *2013**)* Strong inhibition of EV uptake in U87 cells was seen at concentrations as low as 0.1 μg/ml. Culturing these cells with 10% FBS, they showed increased EV aggregation and diminished binding of EVs to the cell surface in the presence of heparin, postulating that these effects were the main cause for uptake inhibition. Binding of the heparin to the EVs and subsequent clustering of the EVs was confirmed by transmission electron microscopy. EVs and heparin both have a negative charge and therefore clustering based on charge-charge interactions is unlikely. EV clustering in presence of heparin is not universal and heparin-based binding assays have been developed to separate heparin-binding and non-heparin-binding EVs.(Zhou et al., 2023) Compared to non-heparin-binding EVs, heparin-binding EVs were enriched in heparan sulfate-binding proteins (HSBP) and histones, and did not express CD81, a common EV marker.(Zhou et al., 2023) Our NTA analysis did not indicate increased aggregation of liposomes in the presence of heparin. As liposomes do not express these biological markers, heparin cannot bind to them and no clustering occurs.

Inhibition of liposome uptake required a thousand-fold higher heparin dose, with significant inhibition observed from 0.1 mg/ml onwards, which also indicates that a different mechanism facilitates uptake inhibition in liposomes compared to EVs.

For our study, we aimed to have a liposome composition similar to previously clinically studied doxorubicin-loaded liposomes,(Ananda et al., 2011) with a PEGylated lipid surface. We used dipalmitoylphosphatidylcholine (DPPC) as our lipid base, as it is a common lipid in liposome formation in general, and used in many (pre-clinical) glioblastoma studies.(Amarandi et al., 2022) DPPC formulated liposomes are slightly negatively charged, which has been shown to be optimal for liposomal uptake in one *in vivo* model.(Ren et al., 2019) Liposomes with different compositions, charges, and sizes may respond different to the presence of heparin, and this is something to be investigated in further studies. Furthermore, while we showed the inhibitory effect of heparin on liposomal uptake in U87, GL261, and Hela cells, more in-depth analysis of different cell lines and conditions is needed.

During incubation in FBS, opsonization of the nanoparticle surface will take place.(Palchetti et al., 2016) Although studies on the nature of the protein corona on the liposome surface are difficult to perform due to the dynamic nature of the corona, its dependence on incubation conditions, and the technological challenges of separating ‘bound’ from ‘unbound’ proteins, it is clear that proteins are adsorbed.(Zanganeh et al., 2017) For several of these (*e.g.* apolipoproteins) cognate receptors exist for which the interaction may be disturbed in the presence of heparin. Anionic nano-particles show less uptake in the presence of serum proteins, whereas cationic nano-particles are not affected.(Fleischer and Payne, 2012) As heparin carries a strong negative charge, it may exacerbate this effect. Incubation with BSA partly restored the inhibitory effect of heparin, thus suggesting that the proteins interact with heparin and the liposomes in some way. However, this could not explain the full effect of FBS, and this interaction needs to be further understood. Second, cancer cells are known to upregulate macropinocytosis to adapt to nutrient-poor environments.(Recouvreux and Commisso, 2017) Uptake was increased overall when cells were cultured without FBS, and this may overcome some of the effect heparin has on uptake.

*In vivo* uptake was inhibited in mice which received clinical doses of heparin before intra-tumor injection of liposomes. We opted to inject these liposomes intra-tumorally to measure the effect of heparin on uptake in tumor cells, not biodistribution. A number of pre-clinical studies showed efficacy of liposomes in delivering compounds to GB,(Lakkadwala et al., 2019; Belhadj et al., 2017; Chen et al., 2017) while clinical trials have thus far failed to improve overall survival.(Ananda et al., 2011; Beier et al., 2009) As it is common for GB patients to receive (low molecular weight) heparin to prevent thrombo-embolic events surrounding surgery or due to diminished mobility, it is likely that some patients in these trials were receiving some form of heparin. Our data suggest that there might be a negative effect of concomitant heparin therapy on the uptake of liposomes in glioma. In this study, our mice received therapeutic dosages of heparin, while patients generally receive prophylactic doses (four times higher) unless specifically indicated. Furthermore, our *in vivo* studies have focused on a subcutaneous model of glioma, which does not take into account the intricacies of biodistribution and the BBB. While previous studies have shown heparin to cross the BBB,(Ma et al., 2002; Li et al., 2017) our study provides a starting point for understanding this interaction rather than a final conclusion. Indeed, more studies will need to be done to fully understand the impact of heparin on the uptake and biodistribution of liposomes in glioblastoma.

## 5 Conclusions

In conclusion, our findings show that heparin interferes with uptake of liposomes in U87 and GL261 glioma cells and that this effect is dependent on the presence of FBS in the media. This is the first study to show uptake inhibition of liposomes by heparin in a subcutaneous tumor model of glioma *in vivo*.

The following are the supplementary data related to this article.

Supplemental movie A: Z-stack imaging of liposome uptake in U87 cells. Liposomes are labeled red with Rhodamine dye. Nucleus stained with DAPI. Cell membrane is green with palmitoylated GFP. Supplementary video 1

Supplementary Figures: uptake of liposomes in vitro and in vivo Supplementary material

## CRediT authorship contribution statement

**Thomas S. van Solinge:** Conceptualization, Methodology, Validation, Formal analysis, Investigation, Writing – original draft, Visualization, Funding acquisition. **Kristina Pagh Friis:** Conceptualization, Methodology, Investigation, Writing – review & editing. **Killian O'Brien:** Methodology, Investigation, Writing – review & editing. **Romy L. Verschoor:** Methodology, Validation, Investigation, Writing – review & editing. **Jeroen van Aarle:** Conceptualization, Methodology, Writing – review & editing. **Arnold Koekman:** Resources, Writing – review & editing. **Xandra O. Breakefield:** Conceptualization, Methodology, Supervision, Funding acquisition, Writing – review & editing. **Pieter Vader:** Conceptualization, Methodology, Resources, Writing – review & editing. **Raymond Schiffelers:** Conceptualization, Methodology, Resources, Writing – review & editing. **Marike Broekman:** Conceptualization, Methodology, Supervision, Funding acquisition, Writing – review & editing, Project administration.

## Declaration of Competing Interest

The authors declare the following financial interests/personal relationships which may be considered as potential competing interests:

Thomas van Solinge reports financial support was provided by Bontius Foundation. Thomas van Solinge reports financial support was provided by Nijbakker-Morra fund. Thomas van Solinge reports financial support was provided by Vrijvrouwe van Renswoude. Thomas van Solinge reports financial support was provided by Bekker-La Bastide Fund. Kristina Friis reports financial support was provided by Alfred Benzon Foundation. Xandra Breakefield reports financial support was provided by National Institutes of Health.

## Data Availability

Data will be made available on request.

## References

[bb0005] Abbott N.J., Patabendige A.A.K., Dolman D.E.M., Yusof S.R., Begley D.J. (2010). Structure and function of the blood-brain barrier. Neurobiol. Dis..

[bb0010] Akbarzadeh A., Rezaei-Sadabady R., Davaran S., Joo S.W., Zarghami N., Hanifehpour Y., Samiei M., Kouhi M., Nejati-Koshki K. (2013). Liposome: classification, preparation, and applications. Nanoscale Res. Lett..

[bb0015] Allijn I.E., Czarny B.M.S., Wang X., Chong S.Y., Weiler M., da Silva A.E., Metselaar J.M., Lam C.S.P., Pastorin G., de Kleijn D.P.V., Storm G., Wang J.W., Schiffelers R.M. (2017). Liposome encapsulated berberine treatment attenuates cardiac dysfunction after myocardial infarction. J. Control. Release.

[bb0020] Amarandi R.M., Ibanescu A., Carasevici E., Marin L., Dragoi B. (2022). Liposomal-based formulations: a path from basic research to temozolomide delivery inside glioblastoma tissue. Pharmaceutics.

[bb0025] Ananda S., Nowak A.K., Cher L., Dowling A., Brown C., Simes J., Rosenthal M.A. (2011). Phase 2 trial of Temozolomide and Pegylated Liposomal Doxorubicin in the treatment of patients with Glioblastoma multiforme following concurrent radiotherapy and chemotherapy. J. Clin. Neurosci..

[bb0030] Atai N.A., Balaj L., van Veen H., Breakefield X.O., Jarzyna P.A., Van Noorden C.J.F., Skog J., Maguire C.A. (2013). Heparin blocks transfer of extracellular vesicles between donor and recipient cells. J. Neuro-Oncol..

[bb0035] Beier C.P., Schmid C., Gorlia T., Kleinletzenberger C., Beier D., Grauer O., Steinbrecher A., Hirschmann B., Brawanski A., Dietmaier C., Jauch-Worley T., Kölbl O., Pietsch T., Proescholdt M., Rümmele P., Muigg A., Stockhammer G., Hegi M., Bogdahn U., Hau P. (2009). RNOP-09: pegylated Liposomal Doxorubicine and prolonged temozolomide in addition to radiotherapy in newly diagnosed Glioblastoma - a Phase II study. BMC Cancer.

[bb0040] Belhadj Z., Zhan C., Ying M., Wei X., Xie C., Yan Z., Lu W. (2017). Multifunctional targeted liposomal drug delivery for efficient Glioblastoma treatment. Oncotarget.

[bb0045] Brandsma D., Kerklaan B.M., Diéras V., Altintas S., Anders C.K., Ballester M.A., Gelderblom H., Soetekouw P.M.M.B., Gladdines W., Lonnqvist F., Jager A., van Linde M.E., Schellens J., Aftimos P. (2014). Phase 1/2A study of Glutathione Pegylated Liposomal Doxorubicin (2B3-101) in patients with brain Metastases (Bm) from solid tumors or recurrent high grade gliomas (Hgg). Ann. Oncol..

[bb0050] Champanhac C., Haas H., Landfester K., Mailänder V. (2021). Heparin modulates the cellular uptake of nanomedicines. Biomater. Sci..

[bb0055] Chen C., Duan Z., Yuan Y., Li R., Pang L., Liang J., Xu X., Wang J. (2017). Peptide-22 and cyclic RGD functionalized liposomes for glioma targeting drug delivery overcoming BBB and BBTB. ACS Appl. Mater. Interfaces.

[bb0060] Crommelin D.J.A., van Hoogevest P., Storm G. (2020). The role of liposomes in clinical nanomedicine development. What now? Now What?. J. Control. Release.

[bb0065] Fleischer C.C., Payne C.K. (2012). Nanoparticle surface charge mediates the cellular receptors used by protein–nanoparticle complexes. J. Phys. Chem. B.

[bb0070] Gabizon A., Shmeeda H., Barenholz Y. (2003). Pharmacokinetics of Pegylated Liposomal Doxorubicin: Review of Animal and Human Studies. Clin. Pharmacokinet..

[bb0075] Gabizon A.A., Patil Y., La-Beck N.M. (2016). New insights and evolving role of pegylated liposomal doxorubicin in cancer therapy. Drug Resist. Updat..

[bb0080] Gaillard P.J., Appeldoorn C.C.M., Dorland R., Van Kregten J., Manca F., Vugts D.J., Windhorst B., Van Dongen G.A.M.S., De Vries H.E., Maussang D., Van Tellingen O. (2014). Pharmacokinetics, brain delivery, and efficacy in brain tumor-bearing mice of Glutathione Pegylated Liposomal Doxorubicin (2B3-101). PLoS One.

[bb0085] Grafals-Ruiz N., Rios-Vicil C.I., Lozada-Delgado E.L., Quiñones-Díaz B.I., Noriega-Rivera R.A., Martínez-Zayas G., Santana-Rivera Y., Santiago-Sánchez G.S., Valiyeva F., Vivas-Mejía P.E. (2020). Brain targeted gold liposomes improve RNAi delivery for Glioblastoma. Int. J. Nanomedicine.

[bb0090] Joshi S., Cooke J.R.N., Chan D.K.W., Ellis J.A., Hossain S.S., Singh-Moon R.P., Wang M., Bigio I.J., Bruce J.N., Straubinger R.M. (2016). Liposome size and charge optimization for intraarterial delivery to Gliomas. Drug Deliv. Transl. Res..

[bb0095] Kasenda B., König D., Manni M., Ritschard R., Duthaler U., Bartoszek E., Bärenwaldt A., Deuster S., Hutter G., Cordier D., Mariani L., Hench J., Frank S., Krähenbühl S., Zippelius A., Rochlitz C., Mamot C., Wicki A., Läubli H. (2022). Targeting immunoliposomes to EGFR-positive Glioblastoma. ESMO Open.

[bb0100] Kolate A., Baradia D., Patil S., Vhora I., Kore G., Misra A. (2014). PEG - a versatile conjugating ligand for drugs and drug delivery systems. J. Control. Release.

[bb0105] La-Beck N.M., Zamboni B.A., Gabizon A., Schmeeda H., Amantea M., Gehrig P.A., Zamboni W.C. (2012). Factors affecting the pharmacokinetics of pegylated liposomal doxorubicin in patients. Cancer Chemother. Pharmacol..

[bb0110] Lakkadwala S., dos Santos Rodrigues B., Sun C., Singh J. (2019). Dual functionalized Liposomes for efficient co-delivery of anti-cancer chemotherapeutics for the treatment of Glioblastoma. J. Control. Release.

[bb0115] Li Y., Sun J.F., Cui X., Mani H., Danner R.L., Li X., Su J.W., Fitz Y., Eichacker P.Q. (2011). The effect of Heparin administration in animal models of sepsis: a prospective study in *Escherichia coli*-challenged mice and a systematic review and metaregression analysis of published studies. Crit. Care Med..

[bb0120] Li H., Chen Y., Deng Y., Wang Y., Ke X., Ci T. (2017). Effects of surface charge of low molecular weight Heparin-Modified cationic liposomes on drug efficacy and toxicity. Drug Dev. Ind. Pharm..

[bb0125] Li J., Tan T., Zhao L., Liu M., You Y., Zeng Y., Chen D., Xie T., Zhang L., Fu C., Zeng Z. (2020). Recent advancements in liposome-targeting strategies for the treatment of gliomas: a systematic review. ACS Appl. Bio. Mater. Am. Chem. Soc..

[bb0130] Li J., Zeng H., You Y., Wang R., Tan T., Wang W., Yin L., Zeng Z., Zeng Y., Xie T. (2021). Active targeting of orthotopic Glioma using biomimetic liposomes co-loaded elemene and Cabazitaxel modified by Transferritin. J. Nanobiotechnol..

[bb0135] Ma Q., Dudas B., Hejna M., Cornelli U., Lee J.M., Lorens S., Mervis R., Hanin I., Fareed J. (2002). The blood-brain barrier accessibility of a heparin-derived Oligosaccharides C3. Thromb. Res..

[bb0140] Ostrom Q.T., Cioffi G., Gittleman H., Patil N., Waite K., Kruchko C., Barnholtz-Sloan J.S. (2019). CBTRUS statistical report: primary brain and other central nervous system tumors diagnosed in the United States in 2012-2016. Neuro-Oncology.

[bb0145] Palchetti S., Colapicchioni V., Digiacomo L., Caracciolo G., Pozzi D., Capriotti A.L., La Barbera G., Laganà A. (2016). The protein corona of circulating PEGylated Liposomes. Biochim. Biophys. Acta Biomembr..

[bb0150] Perry J.R. (2012). Thromboembolic disease in patients with high-grade Glioma. Neuro-Oncology.

[bb0155] Recouvreux M.V., Commisso C. (2017). Macropinocytosis: a metabolic adaptation to nutrient stress in cancer. Front. Endocrinol..

[bb0160] Ren H., He Y., Liang J., Cheng Z., Zhang M., Zhu Y., Hong C., Qin J., Xu X., Wang J. (2019). Role of Liposome size, surface charge, and PEGylation on Rheumatoid Arthritis Targeting therapy. ACS Appl. Mater. Interfaces.

[bb0165] Rouser G., Fleischer S., Yamamoto A. (1969). Two dimensional thin layer chromatographic separation of polar lipids and determination of phospholipids by phosphorus analysis of spots. Lipids.

[bb0170] Siegal T., Horowitz A., Gabizon A. (1995). Doxorubicin encapsulated in sterically stabilized liposomes for the treatment of a brain tumor model: biodistribution and therapeutic efficacy. J. Neurosurg..

[bb0175] Song Z., Huang X., Wang J., Cai F., Zhao P., Yan F. (2021). Targeted delivery of Liposomal temozolomide enhanced anti-glioblastoma efficacy through ultrasound-mediated blood-brain barrier opening. Pharmaceutics.

[bb0180] Stupp R., Mason W., van den Bent M., Weller M., Fisher B., Taphoorn M. Jo (2005). Radiotherapy plus concomitant and adjuvant Temozolomide for Globlastoma. N. Engl. J. Med..

[bb0185] Tannous B.A., Kim D.E., Fernandez J.L., Weissleder R., Breakefield X.O. (2005). Codon-optimized Gaussia Luciferase CDNA for mammalian gene expression in culture and in vivo. Mol. Ther..

[bb0190] Wang H., Zheng M., Gao J., Wang J., Zhang Q., Fawcett J.P., He Y., Gu J. (2020). Uptake and release profiles of PEGylated liposomal doxorubicin nanoparticles: a comprehensive picture based on separate determination of encapsulated and total drug concentrations in tissues of tumor-bearing mice. Talanta.

[bb0195] Xu X., Dai Y. (2010). Heparin: an intervenor in cell communication. J. Cell. Mol. Med..

[bb0200] Zacharias D.A., Violin J.D., Newton A.C., Tsien R.Y. (2002). Partitioning of lipid-modified monomeric GFPs into membrane microdomains of live cells. Science (80).

[bb0205] Zanganeh S., Spitler R., Erfanzadeh M., Alkilany A.M. (2017).

[bb0210] Zhou Y., Yuan R., Cone A.S., Shifflett K.W., Arias G.F., Peng A., Chambers M.G., McNamara R.P., Willcox S., Landis J.T., Pan Y., Griffith J., Dittmer D.P. (2023). Large-scale heparin-based bind-and-elute chromatography identifies two biologically distinct populations of extracellular vesicles. J. Extracell Vesicles.

