# Supplementary figures and images for: Heparin interferes with the uptake of liposomes in glioma
Source: Int J Pharm X. 2023 Jun 21;6:100191. doi: 10.1016/j.ijpx.2023.100191 (PMC10319201; doi:10.1016/j.ijpx.2023.100191)

Supplementary Figure 1

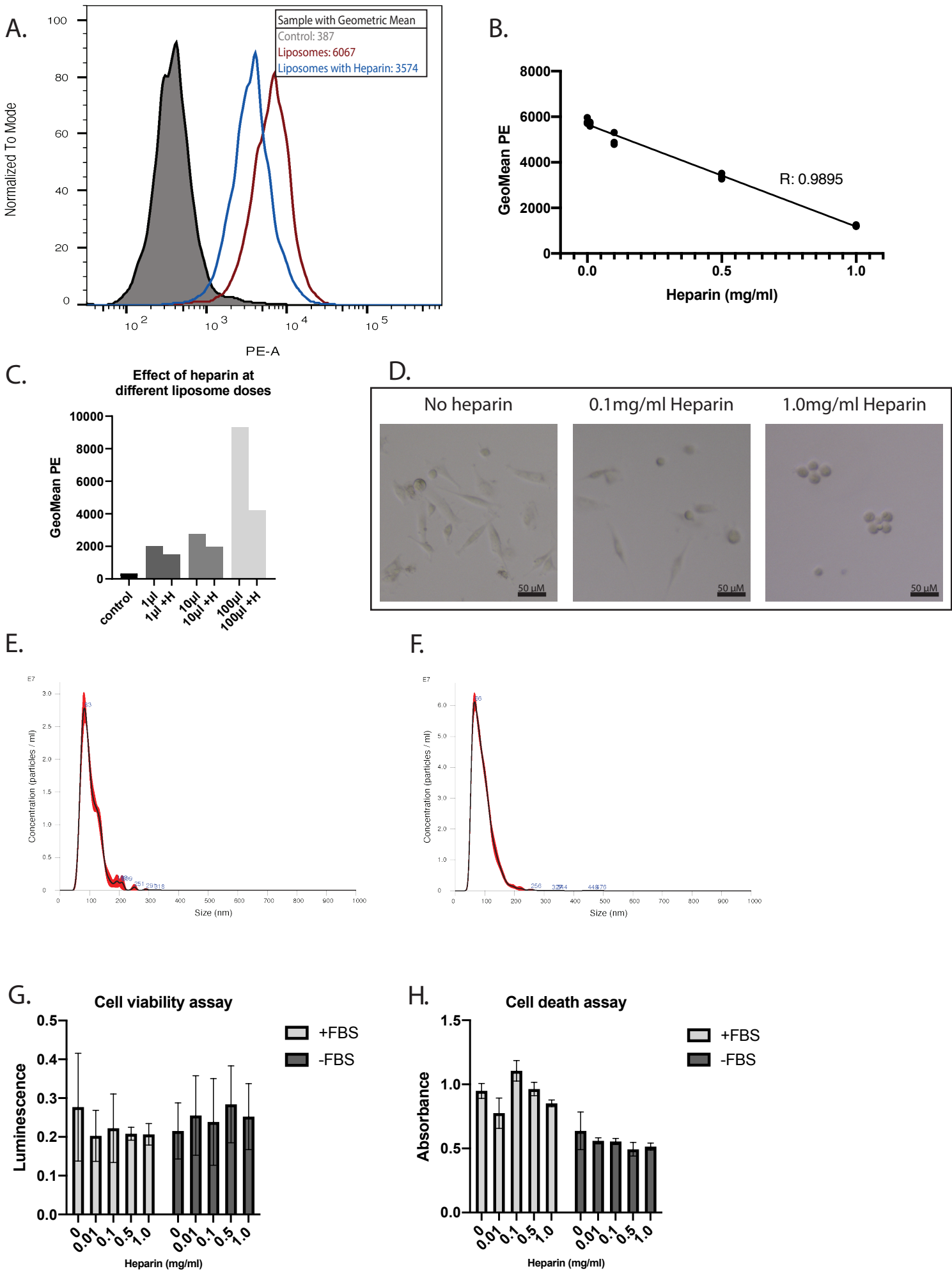

Supplementary Figure 2

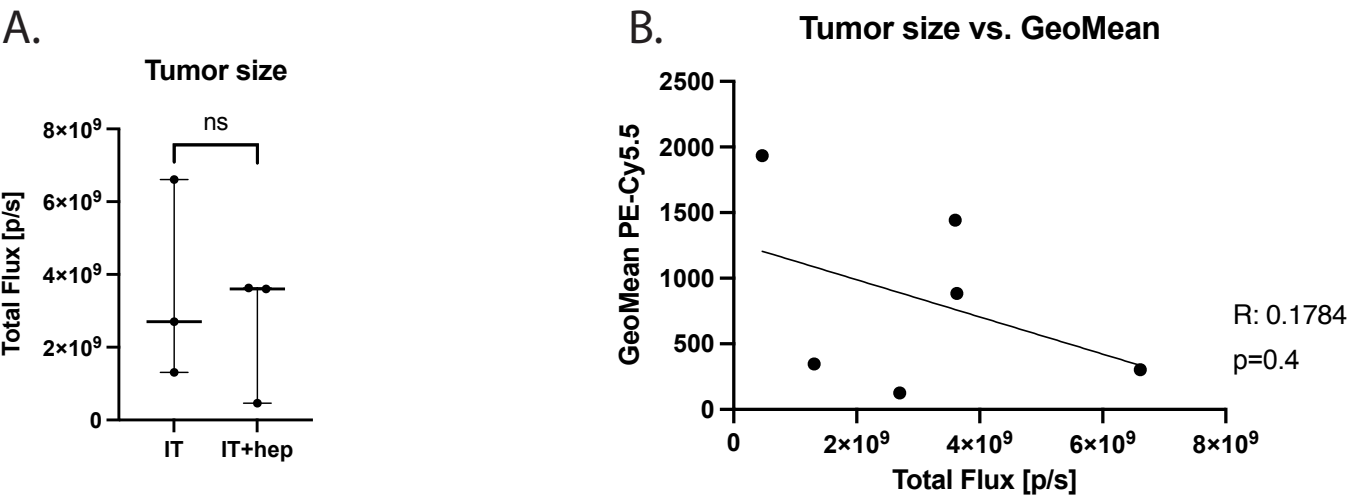

Supplement: Supplementary file 2 — Supplementary Figures: uptake of liposomes in vitro and in vivo [file mmc2.pdf]
